# Repair of Asymmetric Bicuspid Aortic Valve Using Tricuspidation with CardioCel

**DOI:** 10.1093/icvts/ivag044

**Published:** 2026-03-16

**Authors:** Paul P Urbanski, Vadim P Irimie

**Affiliations:** Cardiovascular Clinic, Campus Bad Neustadt, 97616 Bad Neustadt, Germany; Cardiovascular Clinic, Campus Bad Neustadt, 97616 Bad Neustadt, Germany

**Keywords:** aortic valve, bicuspid, reconstruction, valve repair, pericardial patch

## Abstract

We present a novel technique for tricuspidation of the very asymmetrical bicuspid aortic valve (AV) with commissural orientation of about 120-140, via replacement of the fused AV cusp with 2 neo-cusps. The neo-cusps are fashioned from bovine pericardium and sutured to the annulus. Successful repair using the described technique requires good quality and adequate commissure height of the non-fused cusp. Since 2020, this technique has been applied to 15 patients. To date, all patients are alive with well-functioning AVs, presenting no or trivial insufficiency, large orifice areas, and nearly physiological transvalvular gradients.

## INTRODUCTION

An optimal approach for repairing a bicuspid aortic valve (BAV) remains under discussion.^1–5^ Here, we describe a new technique for tricuspidation of the asymmetrical BAV, involving the creation of 2 neo-cusps without any downsizing of the aortic root/annulus.

## SURGICAL TECHNIQUE

The prerequisite for this repair is a very asymmetric BAV phenotype, with commissural orientation of about 120-140°, good quality of the non-fused cusp (NFC), and adequate height of its commissures. Intra-operative transoesophageal echocardiography is mandatory for assessing AV geometry and function, both before and after surgery (**Video 1**). The diameter of the aortic annulus, which is typically larger in BAVs than in tricuspid valves, is not a determining factor, as aortic insufficiency (AI) is primarily caused by a cusp restriction rather than annulus dilatation. Likewise, the height of the commissures of the fused cusp (FC), including the rudimentary commissure between its fused parts, is not relevant because these commissures will be completely recreated correspondingly to the height of the NFC commissures (**Video 2**). In some cases, a kind of chorda is attached at the top of the rudimentary commissure (**Video 2**), as rudimentary remains of the cusp-free margin (FM). After resection of one rudimentary cusp of the FC, the FM of the first neo-cusp is determined with a thick yarn. The yarn reaches from the commissure between the FC and NFC to the middle of the NFC and to the new commissure between 2 neo-cusps. Care should be taken that the top of the neo-commissure (replacing the rudimentary one) should be at the same level as the commissures between the FC and NFC. Corresponding to the determined FM length, a half-round neo-cusp is cut from the bovine pericardium (CardioCel, Admedus Regen Pty Ltd, Malaga WA, Australia). Precise measurement of the patch height prior to suturing the neo-cusp to the AA is not required, as it will be accurately determined during the suturing process. However, the patch height must be greater than the anticipated height (which usually does not exceed 3 cm). The neo-cusp is sutured to the annulus with a 5–0 polypropylene running suture beginning at the nadir of the sinuses and continuing towards the commissures. Thereafter, the remaining rudimentary cusp of the FC is resected, and the FM of the second neo-cusp is determined. The second neo-cusp is sutured to the annulus in the same manner. At the top of the commissures, the sutures are secured with a locked knot at the aortic wall, and the excessive patch material is trimmed along a straight line between the commissures, ensuring the appropriate height of the neo-cusps. The true coaptation height of the cusps is checked with reversed forceps (or, if desired, with any calipers) and, if necessary, additional plications of the FMs are performed with 7–0 polypropylene sutures to adjust the lengths of the FMs of all cusps (all steps described above are demonstrated in **Video 2**). Usually, the suture lines are additionally secured at the tops of the commissures with patch-enforced 6–0 polypropylene U-stitches, passing the neo-cusps and the aortic wall from inside-to-outside (**Video 3**).

**Figure 1.**
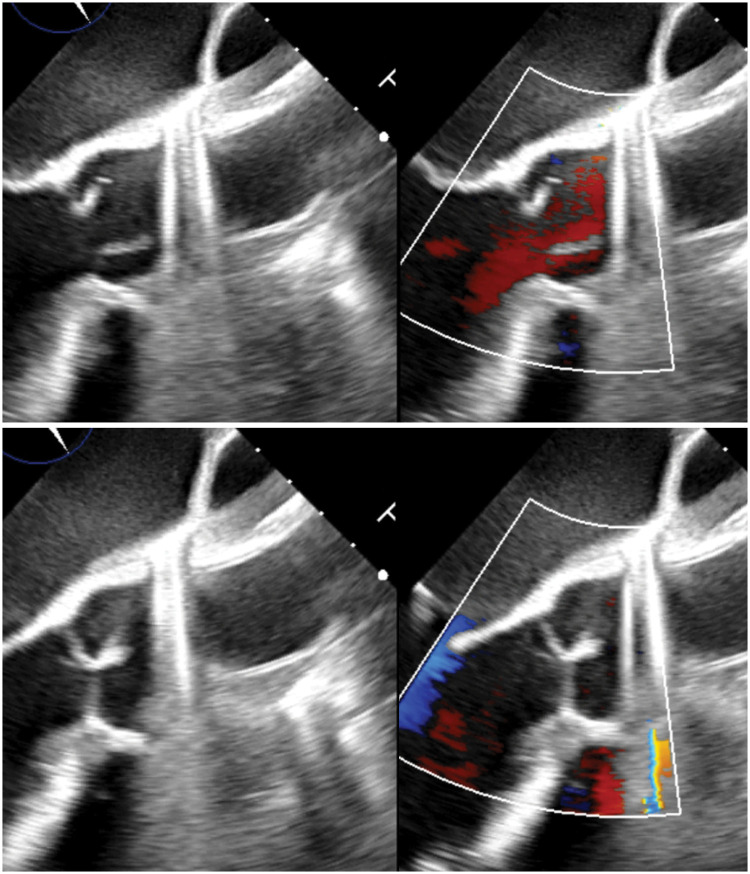
Transoesophageal Echocardiography Demonstrating Opening and Closing of the Reconstructed Bicuspid Aortic Valve by Tricuspidation

Since 2020, this technique has been applied in 15 patients, all of whom are alive with well-functioning AVs (**Figure 1**). During the short- to mid-term postoperative course, no valve-related events were reported. The repaired valves showed excellent haemodynamic characteristics with no or trivial AV-insufficiency in 12 and 3 patients, respectively, and a large geometric orifice area and low mean transvalvular gradient measured at 4.75 cm^2^ (range, 3.6-5.4) and 3.5 mmHg (range, 2-8), respectively.

## DISCUSSION

A repair of asymmetric BAV typically aims for the achievement of both the 180° and the symmetrical orientation of the coaptation line by closing the gap at the end of the incomplete raphe and by moving the commissures towards the symmetric axis of the root.^1–5^ The latter can be achieved, in some degree, without replacing the sinuses;^1^^,^^4^ yet the true BAV symmetry can only be achieved via valve-sparing root repair (VSRR), using a total or partial root-remodelling or valve-reimplantation techniques.^2^^,^^5^ These approaches not only enable the transformation of the commissural orientation to a symmetrical one but also offer the possibility of aortic root repair, should it be necessary for any reason. Taking into consideration that the cause of AI in BAVs is usually a restriction of the cusps (most frequently a restriction of the FC or its rudimentary parts), a downsizing of the aortic root (by performing a VSRR) can improve the cusp coaptation by bringing them closer together. Considering this effect, some authors have proposed a root repair using the valve-reimplantation technique for BAV repair even in patients with normal roots.^2^^,^^6^ However, the concomitant VSRRs, which are inevitable in cases with root pathology, remain questionable in non-pathological roots. A root repair using the reimplantation technique extends the surgery considerably and increases the surgical risk disproportionately, which, in our opinion, cannot be generally justified in patients with non-pathological roots. Additionally, the current ESC/EACTS (2017) and AATS (2018) guidelines do not recommend a root replacement until the root size is 4.5 or 5.0 cm.^7^^,^^8^ Moreover, it must be emphasized that narrowing of the aortic root, particularly of the annulus, can increase the transvalvular gradient, cause functional AV stenosis, and hinder future AV replacement with a sufficiently large valve prosthesis. Yet, an AV-replacement after previous repair should always be kept in mind when repairing BAVs in, usually, relatively young patients who need the largest possible valve prostheses.

**Figure 2.**
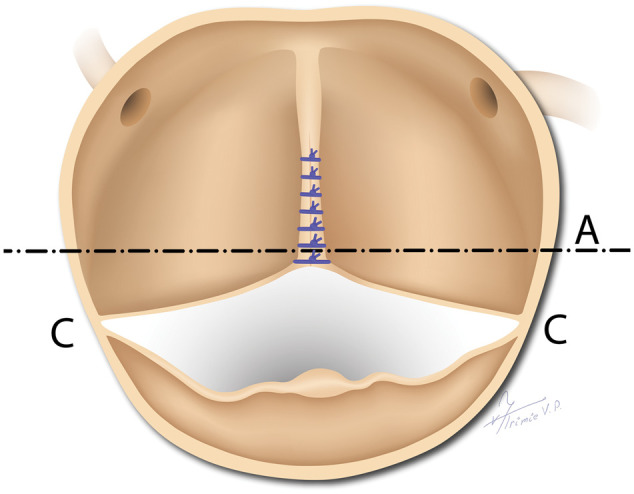
Schematic Illustration of Repaired Asymmetric Bicuspid Aortic Valve. The Repair Consists of the Closure of the Gap at the End of the Short Raphe. Because the Commissures (C) Remain Beyond the Symmetric Axis of the Root (A), the Fused Cusp Cannot Open Sufficiently. See Also Text and References 1 and 5

A similar effect of decreasing the aortic orifice area in asymmetric BAV can result from leaving the FC in place and simply closing the gap at the end of the incomplete raphe (with direct suture or use of a patch material), even if performed without any additional aortic root downsizing. This approach achieves a 180° coaptation line; however, the commissural orientation remains asymmetrical, which may impede optimal opening of the FC due to the shortened FM being positioned beyond the root’s symmetrical axis (see **Figure 2**).

In contrast, the tricuspidation of asymmetric BAVs offers excellent haemodynamic characteristics. Furthermore, the tricuspidation demonstrated here can be performed without any additional annulus downsizing, irrespective of its diameter, which ranged from 26 to 37 mm in our experience. The same regards any additional root downsizing because the described phenotype of BAV is seldom combined with root pathology exceeding the size of 5 cm. In turn, aneurysmal formations of the supracoronary aspect of the aorta (ascending/arch) accompany asymmetric BAVs not infrequently and were approached in 4 of our 15 patients. However, should any root repair be necessary, it can be performed concomitantly to various cusp repairs, including the described tricuspidation.^5^

Even if the patients show good valve competence after this procedure in the short- to mid-term, the durability of this method cannot be determined until the long-term results become available. However, the suitability of CardioCel as a substitute for AV cusps seems to be very promising.^9^ Taking into account that the natural progression of the underlying BAV pathologies is a main cause of limited repair durability,^10^ it remains to be determined which techniques—those that leave the partly degenerated BAV-cusps in place or those with partial cusp replacement—will be more physiological and/or durable. Because the different repairs can impact redo procedures (which, especially after BAV-repairs, are almost unavoidable in younger patients), in various manners, parameters such as operative risk of a reoperation, the size of the implanted AV-prosthesis with postoperative haemodynamics data, and the global survival should always be reported after AV repairs. These data must be taken into consideration to determine the sufficiency of specific repair techniques in selected patients, especially if comparing with standardized AV-replacement.

## Data Availability

The data underlying this article will be shared on reasonable request to the corresponding author.
